# Decline causes of Koalas in South East Queensland, Australia: a 17-year retrospective study of mortality and morbidity

**DOI:** 10.1038/srep42587

**Published:** 2017-02-20

**Authors:** Viviana Gonzalez-Astudillo, Rachel Allavena, Allan McKinnon, Rebecca Larkin, Joerg Henning

**Affiliations:** 1 School of Veterinary Science, Building 8114, The University of Queensland, Gatton, QLD, 4343 Australia; 2 Moggill Koala Hospital, Department of Environment and Heritage Protection, 55 Priors Pocket, Moggill, QLD, 4070 Australia

## Abstract

Koala populations are in catastrophic decline in certain eastern Australian regions. Spanning from 1997–2013, a database derived from wildlife hospitals in southeast Queensland with *N* = 20,250 entries was classified by causes of morbidity and mortality. A total of 11 aetiologies were identified, with chlamydiosis, trauma, and wasting being most common. The clinical diagnosis at submission varied significantly over the observation period. Combinations of aetiologies were observed in 39% of koalas submitted, with chlamydiosis frequently co-occurring. Urogenital (cystitis 26.8%, bursitis 13.5%) and ocular (conjunctivitis 17.2%) chlamydiosis were the most frequently diagnosed representations of the infection. Approximately 26% of submissions comprised koalas involved in vehicle accidents that were otherwise healthy. Age and sex of the koala as well as season and submission period were compared for the case outcomes of ‘dead on arrival’, ‘euthanized’, or ‘released’ for the four most common clinical diagnoses using multinomial logistic regression models. Exploratory space-time permutation scans were performed and overlapping space-time clusters for chlamydiosis, motor vehicle traumas and wasting unveiled high risk areas for koala disease and injury. Our results suggest that these aetiologies are acting jointly as multifactorial determinants for the continuing decline of koalas.

The koala (*Phascolarctos cinereus*) is a folivorous marsupial whose distribution is tied to its food source, the *Eucalyptus* forests in Australia. Eastern Australia has ca. 40% of the ideal habitat for koalas^1^, corresponding to tropical, subhumid and semi-arid regions in the state of Queensland (QLD). Since the 1990s, koalas of South East Queensland (SEQLD) have experienced a severe population reduction. The most recent estimate in the Koala Coast reported a population decline of 80%^2^. Similar population declines experienced in New South Wales (NSW) and Australian Capital Territory (ACT), have contributed to listing the species as “threatened” according to the Australian Federal Environment and Biodiversity Protection Act 2012.

In general, marsupials have declined substantially in Australia: 25% of their original range has been cleared and 24 marsupial species have been declared extinct^3^. As a charismatic animal, the koala faces infectious^4^ and non-infectious^5^,^6^ challenges that need to be closely monitored. In particular, the long-term conservation of the koala is threatened by habitat encroachment and rapid urbanization^7^,^8^. Other threats include overbrowsing of habitat resulting starvation, extreme droughts likely to be linked to climate change and events of stochastic occurrence such as bushfires^9^, trauma related to anthropogenic activities, such as injury and mortality resulting from predation by domestic animals^10^,^11^,^12^ and motor vehicle (MV) collisions^13^. Furthermore inbreeding depression and diseases (mainly by infection with *Chlamydia* spp. and potentially, koala retrovirus- KoRV)^13^,^14^,^15^,^16^ also jeopardize the survival of this species.

Studies aiming at improving our understanding of wildlife diseases are recognized as a powerful way of monitoring ecosystem health and recent developments in host-pathogen ecology, transdisciplinarity, and involvement of endangered species in outbreaks has generated more attention for wildlife health^17^. Public concern and investment in koalas is high, however there are discrepancies in estimated and reported conservation status^18^, which affect overall population management efforts^19^. Despite the listing of koalas as vulnerable to extinction in QLD, NSW and ACT^20^, there is ambiguity regarding QLD population estimates^21^, particularly in inland bioregions. Although the koala distribution in QLD is vast, with its presence being recorded in nine bioregions (total no. 13), population surveys have not covered the entire koala range. Previous reports estimate in QLD koala presence is segmented and rather uncommon, persisting only where ideal habitat occurs^22^. Nevertheless, their occurrence was previously considered common in SEQLD, where there is certainty from surveys conducted on urban koalas^23^ and where current estimates have warned about declining subpopulations since the mid-1990s^14^,^15^. Although this data is difficult to extrapolate to adjacent bioregions as different ecosystems face dissimilar threats, it does provide an overall picture of how future koala conservation and expanding urbanization would interplay.

It is paramount to determine cause-specific morbidity and mortality risk factors to facilitate urgent conservation measures, ensuring the survival and the long-term preservation of the koala. These measures should include preservation of viable habitat, informed local and federal government management policies, and the successful rehabilitation of animals in care. To facilitate this, the aims of the present study are to 1) identify the major causes of morbidity and mortality in SEQLD koalas from 1997–2013; 2) for each cause determine demographic, seasonal and temporal risk factors associated with koalas classified as ‘dead on arrival’, needed to be ‘euthanized’ or ‘released’; and 3) identify spatial-temporal clusters of causes of koala morbidity and mortality.

## Results

### Study population

A dataset of 42,257 records from three hospitals (Moggill Koala Hospital - MKH, Australia Zoo Wildlife Hospital - AZWH, Currumbin Wildlife Sanctuary Hospital - CWSH) in SEQLD spanning from January 1, 1997 through December 31, 2013 was utilized for analysis. A total of *N* = 22,007 koala records were excluded from the analysis to reach a study population of *N* = 20,250. Exclusions included sightings (*N* = 15,113), uncaptured (*N* = 1,044) or instantly released (*N* = 1,127) koalas, relocations (*N* = 650), records with missing information (*N* = 3,234), healthy (*N* = 403), healthy orphaned (*N* = 137) koalas and submissions from states other than QLD (NSW, *N* = 299). Demographically, there were similar numbers of males (*N* = 10,232, 50.9%) and females (*N* = 9,605, 47.8%), and there was no sex information for *N* = 252 (1.25%) records. Adults (*N* = 16,094, 79.5%) were present in much greater proportion than young koalas (*N* = 4,156, 20.5%).

### Aetiologies

A total of 11 aetiologies were identified, which occurred 41,606 times in the 20,250 koalas monitored over the study period. The most common aetiology was *Chlamydia*-like signs (*N* = 21,619, 52.0%), followed by MV trauma (*N* = 6,432, 15.5%), and wasting (*N* = 5,935, 14.3%), amongst others (Table 1).

Within the *Chlamydia*-like signs, we found cystitis to be the most common sign (*N* = 5,422, 26.8%), followed by conjunctivitis (*N* = 3,485, 17.2%), bursitis (*N* = 2,741, 13.5%), pneumonia (*N* = 2,493, 12.3%), nephritis (*N* = 2,432, 12%), and metritis (*N* = 1,022, 5%). Male-specific *Chlamydia*-like signs were diagnosed at a very low frequency overall (e.g. prostatitis *N* = 9, orchitis *N* = 3). ‘Other diseases’ observed included: dermatitis (*N* = 135), septicaemia (*N* = 68), ectoparasitosis (ticks, *N* = 49), and hepatitis (*N* = 47), amongst others. A total of *N* = 900 occurrences of undetermined cause were found.

**Figure 1:**
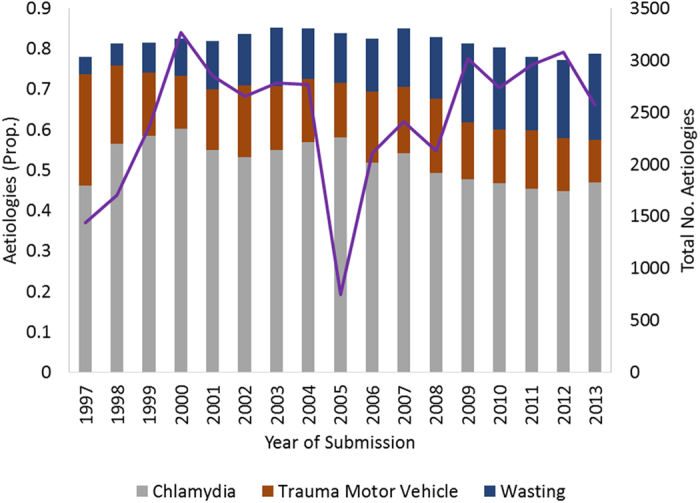
Total counts and proportions of aetiologies *Chlamydia*-like signs, trauma caused by motor vehicles, and wasting occurring in koalas submitted to wildlife hospitals in SEQLD from 1997–2013 (No. of occurrences of aetiologies = 41,606).

There were variations in the occurrences of all aetiologies over time (Fig. 1). The occurrence of *Chlamydia*-like signs has maintained an overall proportion above 30% across all years of the database, despite major variations in nearly two decades. Koalas are being presented less commonly with MV trauma (2009–2013) compared to a decade ago (1997–2001), and current admissions include more emaciated individuals compared to the mid-1990s.

**Figure 2:**
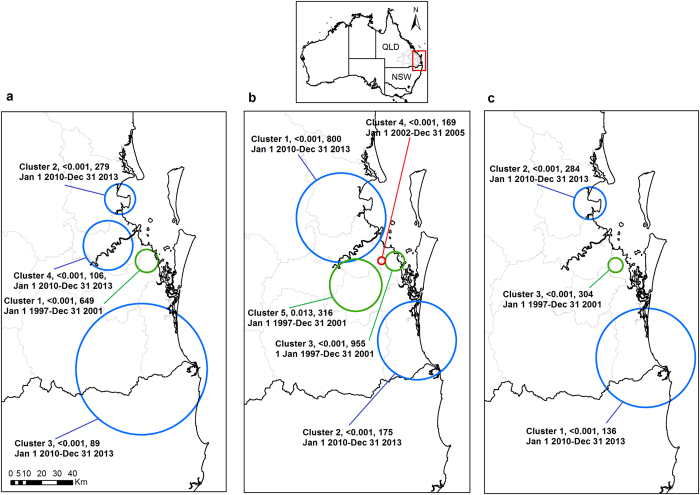
Locations of significant clusters of aetiologies as determined by the space-time permutation scans. (**a**–**c**) The area comprised within the rectangle includes the South East Queensland study area for the space-time permutation. The following map inserts display the significant clusters by the space-time permutation scans: (**a**) trauma by motor vehicle, (**b**) *Chlamydia*-like signs, (**c**) wasting. Each cluster is labelled with its *p*-value, number of observations, and time frame. Maps generated using ESRI ArcGIS Desktop v10.2.1^58^. Centre points for each cluster generated using SaTScan^TM^ v8.0^56^. SaTScan^TM^ is a trademark of Martin Kulldorff. The SaTScan^TM^ software was developed under the joint auspices of (i) Martin Kulldorff, (ii) the National Cancer Institute, and (iii) Farzad Mostashari of the New York City Department of Health and Mental Hygiene.

To identify spatial-temporal clusters of occurrences of aetiologies, we conducted a case-based space-time permutation for the three most prevalent occurrences (*Chlamydia*-like signs, MV trauma, and wasting) for the study period 01/01/1997 to 31/12/2013. Koala cases derived from 345 local government areas grouped within other regions such as the Wide Bay Burnett, Central QLD, and Darling Downs South West, apart from SEQLD, the region from which most observations originated (Table 2). For *Chlamydia*-like signs, the number of locations recorded was 8,170 for 8,331 cases. Five significant *Chlamydia*-like signs clusters were identified. MV trauma was recorded for 4,779 locations totalling 4,998 cases and four significant clusters were identified. Wasting cases were recorded in 4,327 locations comprising of 4,371 cases and three significant clusters were identified. Interestingly, there was an overlap for case-based space-time clusters for occurrences of MV trauma, *Chlamydia*-like signs, and wasting for the 2010–2013 year period (blue clusters in Fig. 2) and for the year period 1997–2001 (green clusters in Fig. 2) and 2010–2013. Additional clusters were observed for *Chlamydia*-like signs for the year periods 1997–2001 and 2002–2005 (Fig. 2b).

### Clinical syndromes

The identified 11 clinical syndromes were diagnosed as a single cause at submission or in combination, hence a total of 159 single or clinical syndrome combinations were made over the study period for the 20,250 records. Clinical syndromes involved single aetiologies in 52.5% (*N* = 10,637), two in 15.8% (*N* = 3,195), three in 13.3% (*N* = 2,914) and 17.3% with four or more aetiologies (*N* = 3,504).

**Figure 3:**
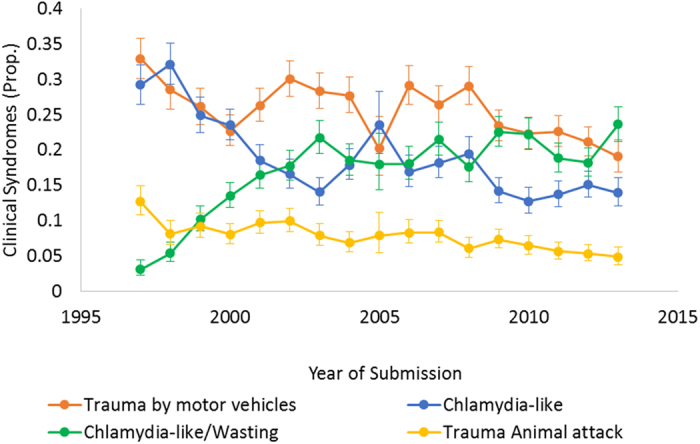
Proportion of top four koala diagnoses (Trauma by motor vehicle, *Chlamydia*-like signs, clinical syndrome combination of *Chlamydia*-like signs & wasting, and trauma by animal attacks) affecting koalas submitted to wildlife hospitals in SEQLD from 1997–2013 with a CI 95% (No. koalas with diagnosis = 20,250).

We focused subsequent analysis on the clinical syndromes with a prevalence >1%, which excluded 14.8% (*N* = 3,007) koalas. MV trauma was the most frequent diagnosis (*N* = 5,183, 25.6%), followed by *Chlamydia*-like signs, and clinical syndrome *Chlamydia*-like signs & wasting (Table 3). Lesser frequency was seen for trauma by animal attacks, trauma by other causes, wasting, and the clinical syndrome combination of *Chlamydia*-like signs & MV trauma, *Chlamydia*-like signs, senescence & wasting, and *Chlamydia*-like sings & trauma by animal attacks. Interestingly, although *Chlamydia*-like signs were the most frequent occurring aetiology (Table 1), it was primarily presented in combination with other aetiologies. On the other hand, submissions due to MV trauma, the leading clinical syndrome, occurred in largely ‘healthy’ animals. The proportion of koalas diagnosed with MV trauma, *Chlamydia*-like signs, and trauma by animal attacks has declined over time, in contrast to the increasing proportion diagnosed with *Chlamydia*-like signs & wasting (Fig. 3).

### Koala outcomes

**Figure 4:**
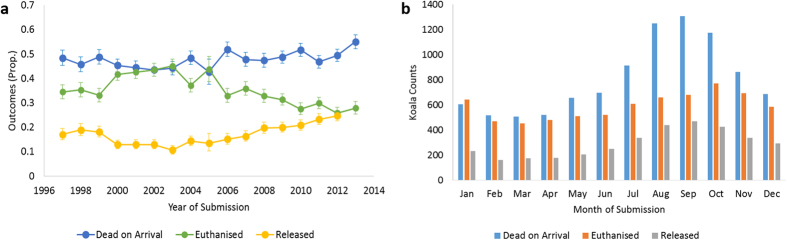
Outcomes (dead on arrival, euthanized, released) for koalas submitted to wildlife hospitals by year of submission between from 1997–2013 (No. koalas with clinical syndromes diagnosed = 20,250). The following figures display the (**a**) Proportion of outcomes by year of submission and (**b**) Counts of outcomes by month of submission.

Most koalas were classified as ‘dead on arrival’ (*N* = 9,690, 47%; CI 95%: 47.2–48.5), followed by ’euthanized’ (*N* = 7,068, 34.9%; CI 95%: 34.3–35.6) and ’released’ koalas (*N* = 3,492, 17.2%; CI 95%: 16.7–17.8). Over time, the proportion of ‘released’ and ‘dead on arrival’ koalas has increased, in contrast to the decreasing trend of euthanized koalas (Fig. 4a). There is also a seasonal pattern influencing koala outcomes (Fig. 4b), largely driven by more dead koalas submitted during August (*N* = 1,249, 6.2%), September (*N* = 1,306, 6.5%), and October (*N* = 1,174, 5.8%), overlapping with QLD’s koala breeding period. There was a slight variation in the proportion of ‘euthanized’ and ‘released’ koalas during this period. Associations of koala-specific (age class, sex) and temporal (year period, season) risk factors with the outcome (‘dead on arrival’, ‘euthanized’ and ‘released’) were explored for the four most frequent clinical syndromes using multinomial logistic regression models. The outcome ‘released’ was used as the reference category.

### Trauma by motor vehicle

MV trauma submissions were at decreased relative risk ratios (RRR) of being ‘dead on arrival’ and ‘euthanized’, compared to released koalas, if submitted between 2005 and 2013 compared to 1997–2001 period (Supplementary Table S1), also highlighted by the higher predicted probability of ‘release’ in those years (Supplementary Figure S1). Adults submitted due to MV trauma were more likely than young koalas to be ‘euthanized’ (compared to ‘released’).

### *Chlamydia*-like signs

A similar temporal trend was observed for koalas submitted with *Chlamydia*-like signs (Supplementary Table S2) and the clinical syndrome combination of *Chlamydia*-like signs & wasting (Supplementary Table S3), although risk of being ‘dead on arrival’ and ‘euthanized’, compared to ‘released’ koalas, declined significantly only during the last four year period (2009–2013). Females with *Chlamydia*-like signs alone or in combination with wasting had higher RRR for being ‘dead on arrival’ or ‘euthanized’ compared to males, also highlighted by the higher predicted probability of ‘release’ for males (Supplementary Figure S2 and S3). Similar to MV trauma, adults submitted with *Chlamydia*-like signs were more likely than young to be ‘euthanized’ than ‘released’.

### Trauma by animal attack

For submissions due to trauma caused by animal attacks, the RRR of being ’euthanized’ or ’dead on arrival’ was significant lower between 2005 and 2013 compared to period 1997–2001 (Supplementary Table S4) – this is similar to MV trauma. Adults compared to young koalas were found to be more likely to be ‘dead on arrival’ and ‘euthanized’ compared to ‘released’, and males were more likely to be ‘euthanized’ compared to ‘released’. Predictive probabilities for this model are shown in Supplementary Table S4.

## Discussion

The current study is the most comprehensive analysis of koala morbidity and mortality to date, and the largest koala database ever explored. Further, this study presents a detailed analysis of factors impacting a specific wildlife population during a time of major population contraction, and serves as a model of other wild species facing extinction events. The koala, although geographically isolated from many other threatened vertebrate species in the world, faces similar perils derived from anthropogenically-induced changes to habitats to which wild species have little time to adapt. The weight of data derived from long-term studies is compelling and aids in the determination of the risks to the future conservation of wild species.

The hospital records analysed here represent a passive surveillance that offers unique opportunities to detect novel or rare pathogens, assist in the establishment of baseline levels of certain diseases, and provide insights into multifactorial causes of injury and disease of wild populations across time and space, and offer a rare opportunity to explore large-scale, multi-seasonal, long-term data that would be otherwise challenging to acquire. This dataset permitted a unique retrospective view into the SEQLD koala populations over a notable period of rapid population decline. Previous passive surveillance studies utilizing hospital records have had considerably smaller sample sizes and had not modelled demographic and temporal risk factors in detail. It would be conceivable to assume wild koalas succumb to these aetiologies at equal rates and this was reflected in the observed hospital submissions.

Trauma was the main cause of admission to SEQLD hospitals, affecting a quarter of admitted koalas that were apparently free of comorbidities. The results of the current study are in agreement with previous studies from SEQLD^24^ and those conducted in other Australian states^5^,^6^,^10^,^11^,^13^. The high proportion of koalas presented to hospitals with traumatic injuries by MV and their high temporal variability may correlate with infrastructural changes across the koala range^25^,^26^, particularly during 2001–2005. Although the total number of submissions varied over time, there was a larger number of koalas excluded in 2005 from the data analysis, due to being submitted as healthy animals or with missing information. Of particular concern is that injured koalas had no underlying disease, which suggests potential for impact on local populations by healthy breeding koalas being prematurely removed. Virtually all koala trauma cases were derived from anthropogenic sources^6^,^10^, directly (MV) or indirectly (dogs, livestock, drowning), except intraspecific aggression. Habitat encroachment due to land clearing and urbanisation, shows a strong association with road vehicle and dog attack trauma. However, residents in these areas may report injured or sick charismatic fauna to hospitals^4^, thus increasing location and selection bias.

Seasonality has been reported^13^ to be a contributing factor to mortality, directly influencing koala behaviour, particularly, dispersal patterns during breeding periods^27^, from August to January in QLD. Although we did not find an association between trauma and seasonality, breeding periods enhance opportunities for sexually-active males to encounter different sources of hazard in search for mates, resources or inbreeding avoidance^24^. Other reports have found young koalas to be more likely to be affected by MV trauma^6^,^28^, potentially triggered by the forced dispersal following weaning^6^ or that the habitat in those sites is fragmented but stable, causing a more natural population dispersal. Dispersal across age classes can be triggered by different drivers across sites^29^; thus, it becomes difficult to pinpoint reasons behind trauma and age class. However, we could speculate that ongoing clearing for development in QLD triggers a uniform age dispersal, enhancing opportunities for all age classes, but as there are more adults present more will encounter roads. In general, there was a higher proportion of adults compared to young koalas in the database, possibly explained by adults comprising a higher population proportion, adults dispersing more readily, are easier to spot, or are removed at a slower rate from the environment by scavengers^29^.

Although previous reports from SEQLD^24^ have found no relationship between injuries caused by animals and sex, the demographics of trauma by animal attacks can reflect koala biological traits. Mortality surveys from other states^11^,^30^ have determined that the majority of attacks by domestic carnivores occur in koalas with poor body condition, suggesting an increased vulnerability to trauma when debilitated as they roam more frequently on the ground. This finding contrasts with the present study, in which the top two traumatic injuries (MV and dogs) occurred in apparently healthy individuals. Although detailed post-mortem and histological examinations that would exclude any underlying disease were not performed in every dead individual, an ongoing prospective mortality survey conducted by our research team indicates most koalas affected by trauma are healthy (unpublished data). Overall, trauma by animal attacks displays a slight but steady decrease across the years, which may reflect the efforts from the QLD State sponsored Koala Plan^31^ to identify threats, incorporate careful planning of koala sensitive areas in development, and educate regarding responsible pet ownership^15^. In general, the ‘released’ koala proportion for all clinical syndromes has increased, suggesting increased success in rehabilitation efforts. Additional threat mitigation efforts currently under consideration or being carried out to aid include differential speed signs^25^, installation of Intelligent Transportation System devices for driver awareness^32^,^33^, dog registrations^10^, to cite a few. Additional traffic-calming measures such as daylight savings^34^ can be adopted in QLD to decrease road-associated injury and mortality, following careful consideration of its impact on diurnal species. These measures could aid in the alleviation of current road and dog associated mortality for wild crepuscular or nocturnal species in Australia and elsewhere.

Risk factor analysis for trauma by other causes (e.g. drowning, intraspecific aggression, tree falls - data not shown here) highlight that younger koalas were at higher risk for being submitted ‘dead on arrival’. One plausible explanation is that young and inexperienced koalas may suffer lethal trauma following falls whilst climbing or during fights^13^. Adults are perhaps more likely to survive misadventure, arriving to hospitals with terminal injuries, resulting in euthanasia.

The relationship of koala population decline with disease has been less clear. Pathogens relying on frequency-dependent transmission such as *Chlamydia* spp. can influence population dynamics^35^, affecting koalas by increasing mortality from wasting and blindness and decreasing population recruitment through impairment of reproduction^36^. However, common diseases such as chlamydiosis and potentially KoRV-driven immunodepression seem to only play an important role at the population when other extrinsic factors inducing physiologic stress^37^, stemming from urbanisation such as habitat fragmentation^22^,^25^,^37^ are present. This is particularly interesting with KoRV, as there is a 100% prevalence of this endogenous virus in QLD koalas^38^; however, according to this study, the leading submission causes do not correspond to immunosuppressive disease, lymphoma, or leukaemia which are thought to be linked to KoRV-B infection^39^.

*Chlamydia*-like signs made up the second and third (in combination with wasting) largest groups of submissions, which agrees with past reports^6^. *Chlamydia* can affect multiple body systems, leading to chronic illness and euthanasia on welfare grounds. In koalas, chlamydial infection is a disease complex represented by four syndromes (rhinitis-pneumonia, ocular, reproductive, urinary tract infection). Immunohistochemistry for *Chlamydia* has demonstrated positive labelling for other sites such as the rectum, cloaca, spleen and lung, demonstrating a multisystemic spread of the infectious organism^40^. The two species known to date to infect koalas are *C. pneumoniae* and *C. pecorum*^41^. *C. pecorum* has been isolated from ocular, urogenital and rectal sites, and is commonly associated with overt clinical disease and thus thought to be more virulent^36^. The usual site for infection for *C. pneumoniae* is the ocular system; with infection at other sites infrequent^36^,^41^. Briefly, cystitis, or ‘wet bottom’, a primary cause of submission in other states^6^, leads to other ascending urogenital disease manifestations^40^,^41^ further compromising multiple organ systems^41^. Ovarian bursitis and orchitis cause infertility^24^,^41^,^42^,^43^,^44^,^45^ and although they may not cause direct mortality, bursitis is a reason for euthanasia due to infertility according to QLD governmental guidelines. Chlamydial urogenital disease continues to be the most prevalent chlamydiosis manifestation^10^,^11^,^30^,^46^,^47^, likely contributing to a gradual deterioration, in turn increasing detection in comparison to other diseases or chlamydial manifestations, leading to overrepresentation. Conjunctivitis leads to blindness and thus increased predation and starvation due to affecting foraging capacity. Other signs such as the rhinitis-pneumonia complex have been reported previously in higher numbers^46^; we report a declining proportion for nephritis and pneumonias. Lower proportion of pneumonias correlates with observations reported by MKH, which vaccinated incoming vulnerable (e.g. orphaned) koalas in previous years (pers. comm. P. Theilemann).

Studies in free-ranging koalas in QLD have reported variability in prevalence of chlamydiosis in the wild^36^, and the existence of healthy carriers, suggesting that infection prevalence does not equal manifestation of disease. Subclinical infections may explain the underrepresentation of chlamydiosis in our study in comparison to trauma-associated cases, particularly as road-killed koalas will be invariably more visible to the public than koalas affected by chlamydiosis or other chronic, debilitating disease associated with forested settings. Another source of underrepresentation of chlamydiosis may be due to healthy carriers particularly if infected with less virulent species (e.g. *C. pneumoniae*^36^). The demographics of chlamydiosis diagnoses will likely shift in the future in response to the introduction of more accurate, cost-effective diagnostic tools^42^.

For some causes, particularly chlamydiosis, the female sex was a risk factor associated with poorer clinical outcomes, highlighting the impact of the disease on the female koala population in hospitals. Most studies have focused on female reproductive pathology from the standpoint of burden of disease and infertility sequelae^48^. Female chlamydiosis is diagnosed more readily due to overt disease expression, leading to poor clinical outcomes as infertile individuals are euthanased. Further, the increased incidence in females maybe a confounder derived from the active surveillance performed with ultrasound on females, masking and subsequently underdiagnosing the less-evident male reproductive disease.

Acute or chronic chlamydiosis results in poor prognosis for SEQLD koalas, particularly if combined with wasting. Progressive wasting, or emaciation in a wild koala can be due to chronic debilitation due to disease^10^ or dental attrition caused by advanced age. Adults are more likely to be wasted due to cumulative risk of exposure to pathogens, sources of injury, dental attrition from advanced age and environmental stressors, compared to young koalas. Dental attrition jeopardizes the koala’s masticatory effectiveness, leading to progressive body condition loss as for hindgut fermenters, food particle size and cell wall breakdown is necessary to maximize microbial fermentation^49^. However, most submitted koalas were not senescent, indicating the increased proportion of wasted koalas in QLD over time is due to undiagnosed chronic diseases. Further research, with detailed post mortem examinations will help elucidate the causes of the increased proportion of wasting, and add to the weight of data in regards to the associations between the different aetiologies and outcomes found in this study. Similar efforts conducted in other states with comparable population structure, pressures, climate and geography (NSW)^50^ paired with continuing studies addressing aspects of dynamics of the SEQLD population are needed to project conclusions concerning the future of contracting koala populations. Findings from previous post-mortem surveys report virtually the same frequency and type of aetiologies^5^,^10^,^11^,^24^, with minor variations concerning certain diseases, which indicates that the pressures that koalas face across their range have remained the same across space and time, are unlikely to change in the near future, and are likely capable of causing local extinctions^23^.

Besides chlamydiosis, there were also highly variable temporal patterns in other less frequent disease manifestations, highlighting the overall variability of different aetiologies across time. The ‘other diseases’ category included dermatitis, septicaemia, ectoparasitosis, hepatitis, amongst others; however, their significance in SEQLD koala subpopulations could not be discerned in the present study.

In the exploratory spatial analysis overlapping clusters between MV trauma, *Chlamydia*-like signs, and wasting were identified for two year periods (2010–2013 and 1997–2001). The fact that wasting and *Chlamydia*-like signs cases were observed in the same spatial-temporal regions could unveil clusters in SEQLD related to undiagnosed infectious disease. The intense, long-term habitat clearing in QLD^25^,^26^,^51^ could be leading to starvation in koalas, an issue that has surprisingly not generated much discussion. Most of these clusters also are located within densely populated councils. Thus, this could reflect (1) a direct observant effect from increased numbers of reporting residents; or (2) a true disease (stressed koalas expressing overt disease) and injury (higher traffic or road density) cluster derived from increased risk. Future spatial-temporal modelling would be required to identify environmental and climatic risk factors for morbidity and mortality.

Medical records or passive surveillance data for research is an increasingly attractive area, particularly for wildlife investigations, offering a rare and cost-effective opportunity to explore large-scale, multi-seasonal, long-term data that would be otherwise challenging to acquire. Unlike with active surveillance, some bias is expected with passive surveillance as it does not comprise specific standardised data collection to assess populations for evidence of disease. For example, people may be more likely to report a koala than the more commonly-urbanized Australian possums (taxonomic bias) or adults and males would be easier to spot than young koalas (demographic bias). Nonetheless, this data set is valuable due to the large number of records over time and space. Some dead koalas submitted to hospitals receive no or cursory necropsies due to financial constraints, limited staff time or expertise, potentially leading to mis- or underdiagnosis of true causes of mortality and morbidity, and failure to identify comorbidities. Thus data extracted from hospitals or rehabilitation centres are inherently flawed (e.g. misdiagnosis due to lack of validated testing in certain pathological conditions; varying public engagement attitudes towards wildlife in a certain areas; lack of consistency in post-mortem assessments, submission of carcasses with limited diagnostic value due to a suboptimal preservation, etc.). However, these institutions remain an important source of passive surveillance data that would be otherwise challenging to actively collate.

Our analysis permitted a unique retrospective view into the SEQLD koala populations over a notable period of rapid population decline, offering the possibility to infer prospective dynamics and risk factors. Results may serve to determine risk factors in other wild species impacted in this geographic area and more broadly by urbanisation threats, allowing for a holistic wildlife management and conservation policy to be developed in threatened species.

This is the first comprehensive study to analyse retrospectively the complex nature of the koala decline in the SEQLD, spanning nearly two decades. Future directions needed to curb the ongoing decline of koalas in QLD and adjacent states and territories include: reduction of euthanasia rates, further research to identify environmental risk factors of morbidity and mortality, and development of science-based mitigation strategies including instating restricted clearing for development, building road capacity, encouragement of responsible pet ownership, standardised national surveys and detailed mortality studies.

## Methods

### Study population

Data for this study originated from clinical database managed by the MKH, which has been under the supervision of the Department of Environment and Heritage Protection (DEHP) since 1991. In this database, sightings and submissions of koalas to wildlife hospitals are recorded. Sightings are defined observations of free-ranging koalas made by the public or rangers, but that were never admitted to the hospital. The MKH receives submissions from the public encompassing an area of 349,729 km^2^ in Queensland, grouped by local government areas (e.g. councils). Two other major wildlife hospitals in SEQLD (AZWH and CSWH, amongst others) report their koala submissions to MHK. Data entry in the clinical database commenced in 1997. In 2014, the clinical database, originally developed in Microsoft Access 2007, was converted into a state-of-art online SQL database called ‘KoalaBASE’, with significantly improved data entry and reporting features (http://www.ava.com.au/node/27181).

For submitted individuals, upon reception, mandatory information is gathered in a record sheet, including the exact koala collection location, demographic data, and previous identification information (if any). A physical examination is conducted, in which any clinical signs, injuries and identification features, including microchip number are noted. Koalas are aged according to the wear of the last pre-molar and first molar^52^, or by identifying the individual with previous submissions. Any koalas that were rehabilitated were microchipped prior to release. Sightings, uncaptured, or captured and instantly released, relocated, submissions with missing information, healthy, healthy orphans, and koalas from states other than QLD were excluded from the analysis.

### Quantitative analysis

Data mining was performed using Microsoft Excel^®^ 2013. Re-categorization of outcome (‘dead on arrival’, ‘euthanized’, ‘released’) and aetiology of submission into pre-determined categories was necessary prior to quantitative analysis. A classification chart for the following aetiological causes was developed: (1) MV Trauma, (2) *Chlamydia*-like signs, (3) *Chlamydia*-like signs & Wasting, (4) Trauma by animal attack, (5) *Chlamydia*-like signs & MV trauma, (6) Undetermined, (7) Trauma by other causes, (8) *Chlamydia*-like signs & Senescence & Wasting, (9) *Chlamydia*-like signs & Trauma by animal attack, (10) Wasting and (11) Other.

### Koala submissions

Koala submissions were then classified in two ways, (a) aetiologies (b) clinical syndromes. An ‘aetiology’ describes a discrete (single) cause of submission of a koala to a hospital and is used to quantify the overall frequency of aetiologies, thereby highlighting the temporal and spatial importance of individual aetiologies.

Since koalas can be exposed to several aetiologies, multiple occurrences of different aetiologies can be recorded for the same koala (e.g. a koala can have occurrences of the aetiology ‘*Chlamydia’* and of the aetiology ‘Trauma’). Therefore the term ‘clinical syndrome’ summarizes what ultimately caused the disease/injury and death of koalas (which could be a single aetiology or a combination of aetiologies). The term ‘clinical syndrome’ is used to describe the frequency of the conditions that lead to the disease/injury and death of koalas and to model risk factors associated with outcomes (‘dead on arrival’, ‘euthanized’, ‘released’) for each diagnosis.

Data analysis was conducted using the software package STATA v. 14.0 (StataCorp, USA). Data were arranged in a contingency tables to describe the frequency of occurrences of aetiologies, clinical syndromes and outcome (‘dead on arrival’, ‘euthanized’, ‘released’) across koala-specific (age class, sex) and temporal (year period, season) explanatory variables. Explanatory variables were categorised as following: dichotomized age [young (baby or joey, young and subadult), adult], sex [female, male], season [January-March, April-June, July-September, October-December], and submission year periods [1997–2001, 2002–2005, 2006–2009, 2010–2013]).

We explored the association of koala-specific and temporal risk factors with the outcome (‘dead on arrival’, ‘euthanized’ and ‘released’) for the four most frequent clinical syndromes using multinomial logistic regression models. ‘Released’ koalas were considered the base category used for comparison for koalas ‘dead on arrival’ and koalas that were ‘euthanized’. Approximately 2% of records had missing risk factor information had to be excluded from the analysis. A multiple Wald test was computed to evaluate the statistical significance of all categories together for any categorical risk factor variable^53^. Variables for which *p* < 0.10 in the univariate analysis were considered for multivariable analysis.

A backward and forward model selection process was run, and *p*-values, RRR with 95% confidence intervals for each explanatory variable were calculated. The stepwise selection process was stopped once all covariates were significantly (*p* < 0.05) contributing to the model. First order interactions between explanatory variables were also explored. Only main effects were tested and explanatory variables significant at *p* < 0.05 were maintained. If a variable was not selected for the initial multivariable model, it was added back to the model, and significant variables (if so) were retained. This process allowed the identification of explanatory variables that may not influence the outcome by themselves, but contribute significantly to it when combined with other explanatory variables^54^. The Bayesian information criterion was used compare to non-nested models for the same clinical syndrome. Goodness-of-fit statistics were calculated to assess how well the final model fit the data. Predicted probabilities for each outcome were calculated using the ‘margins’ command in STATA, holding the remaining variables in the model at their means.

A case-based space-time permutation model^55^ using Kulldorff’s SaTScan™^56^ software was developed to identify spatial-temporal clustering or ‘hotspots’ of occurrences of aetiologies. Since population-at-risk data was not available, a large number of random permutations of the spatial and temporal attributes of each koala case in the dataset was produced to calculate the scan statistics. Most likely clusters were calculated for each simulated dataset and statistical significance was evaluated using Monte Carlo hypothesis testing^57^.

## Additional Information

**How to cite this article**: Gonzalez-Astudillo, V. *et al*. Decline causes of Koalas in South East Queensland, Australia: a 17-year retrospective study of mortality and morbidity. *Sci. Rep.* **7**, 42587; doi: 10.1038/srep42587 (2017).

**Publisher's note:** Springer Nature remains neutral with regard to jurisdictional claims in published maps and institutional affiliations.

## Supplementary Material

Supplementary Material Tables

## Figures and Tables

**Table 1:** Frequency of aetiologies of submission (with a CI 95%) in koalas submitted to wildlife hospitals from 1997 through 2013 (No. of occurrences of aetiologies = 41,606).

Aetiologies	Frequency	Proportion
*Chlamydia*-like signs	21,619	52.0
Trauma Motor Vehicle	6,432	15.5
Wasting	5,935	14.3
Trauma Animal Attack	2,154	5.2
Trauma other causes	1,170	2.8
Senescence	1,160	2.8
Other diseases	1,097	2.6
Undetermined	900	2.1
Cancer	420	1.0
Blind	678	1.6
Congenital/Hereditary	41	0.1
Total	41,606	100

**Table 2:** Details of significant clusters of aetiologies as determined by the space-time permutation scans.

Aetiologies	Time frame	Cases	Expected	No. LGA	Council epicentre	LGA epicentre	Diameter (Kms.)	*p-*value
Trauma motor vehicle	1997/1/1 to 2001/12/31	649	445	14	Redland City	Sheldon-Mt Cotton	7.4	<0.001
	2010/1/1 to 2013/12/31	279	155	15	Moreton Bay Regional	Rothwell-Kippa-Ring	9.9	<0.001
	2010/1/1 to 2013/12/31	89	30	40	Gold Coast City	Guanaba-Springbrook	42.4	<0.001
	2010/1/1 to 2013/12/31	106	54	145	Brisbane City	Toowong	16.0	<0.001
*Chlamydia*-like signs	2010/1/1 to 2013/12/31	800	438	145	Moreton Bay Regional	Central Pine West	28.9	<0.001
	2010/1/1 to 2013/12/31	175	46	38	Gold Coast City	Broadbeach Waters	25.4	<0.001
	1997/1/1 to 2001/12/31	955	724	10	Redland City	Thornlands	6.2	<0.001
	2002/1/1 to 2005/12/31	169	108	3	Brisbane City	Burbank	2.4	<0.001
	1997/1/1 to 2001/12/31	316	245	55	Logan City	Greenbank-Boronia Heights	16.9	0.013
Wasting	2010/1/1 to 2013/12/31	136	52	38	Gold Coast City	Mudgeeraba-Reedy Creek	32.1	<0.001
	2010/1/1 to 2013/12/31	284	170	15	Moreton Bay Regional	Rothwell-Kippa-Ring	10.4	<0.001
	1997/1/1 to 2001/12/31	304	195	7	Redland City	Sheldon-Mt Cotton	4.7	<0.001

Koalas were submitted with trauma by motor vehicle, *Chlamydia*-like signs and wasting to wildlife hospitals in South East Queensland, from 1997 through 2013 (No. koalas with trauma by motor vehicle, *Chlamydia*-like signs and wasting and spatial information = 15,524).

**Table 3:** Frequency of the top 10 (>1%) clinical syndromes in koalas submitted to wildlife hospitals in SEQLD from 1997 through 2013 (No. koalas with diagnosis at prevalence of >1% = 17,243).

Clinical Syndromes	Frequency	Proportion
Trauma motor vehicle	5,183	25.6
*Chlamydia*-like signs	3,744	18.5
*Chlamydia*-like signs-Wasting	3,483	17.2
Trauma animal attack	1,561	7.7
*Chlamydia*-like signs-Trauma motor vehicle	811	4.0
Undetermined	728	3.6
Trauma other causes	686	3.4
*Chlamydia*-like signs-Senescence-Wasting	517	2.5
*Chlamydia*-like signs-Trauma animal attack	271	1.3
Wasting	259	1.3
	17,243	100
