# Supplementary material for: Decline causes of Koalas in South East Queensland, Australia: a 17-year retrospective study of mortality and morbidity
Source: Sci Rep. 2017 Feb 20;7:42587. doi: 10.1038/srep42587 (PMC5316976; doi:10.1038/srep42587)

Decline causes of Koalas in South East Queensland, Australia: a 17-year retrospective study of mortality and morbidity

Viviana Gonzalez-Astudillo[1]*, Rachel Allavena[1], Allan McKinnon[2], Rebecca Larkin[2], and Joerg Henning[1].

[1]School of Veterinary Science, Building 8114, University of Queensland, Gatton, QLD, 4343 Australia

[2]Moggill Koala Hospital, Department of Environment and Heritage Protection, 55 Priors Pocket, Moggill, QLD, 4070 Australia

*v.gonzalez@uq.edu.au

Supplementary Material Table S1. Trauma by motor vehicles (*N*=5,047): Final multinomial logistic regression of risk factors associated with outcome of diagnosis (dead on arrival, euthanized, released) for koalas submitted to wildlife hospitals in SEQLD from 1997-2013 (reference category: *released* koalas).

		Dead on Arrival				Euthanised			
**Risk Factor**	***N* koalas submitted**	**Percentage**	**RRR (CI 95%)**	***p***	**Wald test**	**Percentage**	**RRR (CI 95%)**	***p***	**Wald test**
**Year Period**									
1997-2001	1,548	69.6	1		<0.001	16.3	1		<0.001
2002-2005	1,115	73.4	1.27 (1.00-1.60)	0.050		14.9	1.11 (0.83-1.49)	0.473	
2006-2009	1,259	69.7	0.76 (0.62-0.93)	0.008		11.7	0.54 (0.41-0.71)	0.000	
2010-2013	1,125	70.0	0.66 (0.54-0.81)	<0.001		8.6	0.34 (0.25-0.45)	<0.001	
**Age Class**									
Young	1,466	73.3	1			-	9		1
Adult	3,581	69.5	1.07 (0.91-1.27)	0.392		14.7	1.83 (1.44-2.33)	<0.001	

RRR – Relative Risk Ratio
CI – Confidence Intervals

Supplementary Material Table S2. *Chlamydia*-like signs (*N*=3,730): Final multinomial logistic regression of risk factors associated with outcome of diagnosis (dead on arrival, euthanized, released) for koalas submitted to wildlife hospitals in SEQLD from 1997-2013 (reference category: *released* koalas).

		Dead on Arrival				Euthanised			
**Risk Factor**	***N koalas submitted***	**Percentage**	**RRR (CI 95%)**	***p***	**Wald test**	**Percentage**	**RRR (CI 95%)**	***p***	**Wald test**
**Year**									
1997-2001	1,474	20.0	1		<0.001	48.5	1		<0.001
2002-2005	684	13.0	0.52 (0.39-0.69)	<0.001		52.0	0.83 (0.66-1.03)	0.096	
2006-2009	826	25.4	0.90 (0.71-1.14)	0.394		36.8	0.51 (0.41-0.64)	<0.001	
2010-2013	746	20.6	0.41 (0.32-0.52)	<0.001		20.1	0.14 (0.11-0.18)	<0.001	
**Sex**									
Female	2,068	21.3	1			55.1	1		
Male	1,642	18.5	0.31 (0.26-0.38)	<0.001		23.3	0.14 (0.11-0.16)	<0.001	
**Age**									
Young	575	24.0	1			25.7	1		
Adult	3,155	19.5	1.22 (0.96-1.54)	0.099		44.0	2.67 (2.11-3.39)	<0.001	

Supplementary Material Table S3. *Chlamydia*-like signs & Wasting (*N*=3,477): Final multinomial logistic regression of risk factors associated with outcome of diagnosis (dead on arrival, euthanized, released) for koalas submitted to wildlife hospitals in SEQLD from 1997-2013 (reference category: *released* koalas).

		Dead on Arrival				Euthanised			
**Risk Factor**	***N koalas submitted***	**Percentage**	**RRR (CI 95%)**	***p***	**Wald test**	**Percentage**	**RRR (CI 95%)**	***p***	**Wald test**
**Year**									
1997-2001	610	32.0	1		<0.001	66.6	1		<0.001
2002-2005	781	24.8	0.60 (0.25-1.45)	0.256		73.5	0.83 (0.35-1.97)	0.674	
2006-2009	981	36.2	0.93 (0.40-2.17)	0.861		62.3	0.74 (0.32-1.71)	0.475	
2010-2013	1,105	47.8	0.22 (0.11-0.44)	<0.001		44.5	0.09 (0.04-0.18)	<0.001	
**Sex**									
Female	1,927	35.1	1			62.9	1		
Male	1,543	38.4	0.32 (0.21-0.48)	<0.001		56.2	0.23 (0.15-0.34)	<0.001	
**Age**									
Young	203	43.3	1			49.7	1		
Adult	3,274	36.2	2.17 (1.17-4.02)	0.014		60.5	3.42 (1.83-6.39)	<0.001	

Supplementary Material Table S4. Trauma by animal attacks (*N*=1,537): Final multinomial logistic regression of risk factors associated with outcome of diagnosis (dead on arrival, euthanized, released) for koalas submitted to wildlife hospitals in SEQLD from 1997-2013 (reference category: *released* koalas).

		Dead on Arrival			Euthanised				
**Risk Factor**	***N koalas submitted***	**Percentage**	**RRR (CI 95%)**	***p***	**Percentage**	**Wald test**	**RRR (CI 95%)**	***p***	**Wald test**
**Year**									
1997-2001	546	64.1	1		21.4	<0.001	1		<0.001
2002-2005	329	62.6	0.94 (0.63-1.40)	0.773	22.5		1.03 (0.65-1.64)	0.905	
2006-2009	361	56.2	0.42 (0.30-0.59)	<0.001	14.1		0.32 (0.21-0.50)	<0.001	
2010-2013	301	55.8	0.40 (0.28-0.57)	<0.001	14.6		0.29 (0.18-0.47)	<0.001	
**Age**									
Young	606	60.2	1		14.7		1		
Adult	931	60.4	1.42 (1.09-1.85)	0.010	21.2		2.01 (1.42-2.83)	<0.001	
**Sex**									
Female	689	61.2	1		16.1		1		
Male	838	59.4	1.13 (0.87-1.46)	0.376	20.6		1.45 (1.04-2.02)	0.030	

Supplementary Material Figure S1. Trauma by motor vehicle: Predicted probabilities (with 95% confidence intervals) derived from multinominal regression models of risk factors associated with the diagnostic outcomes (dead on arrival, euthanized, released) for koalas submitted to wildlife hospitals in SEQLD from 1997-2013.

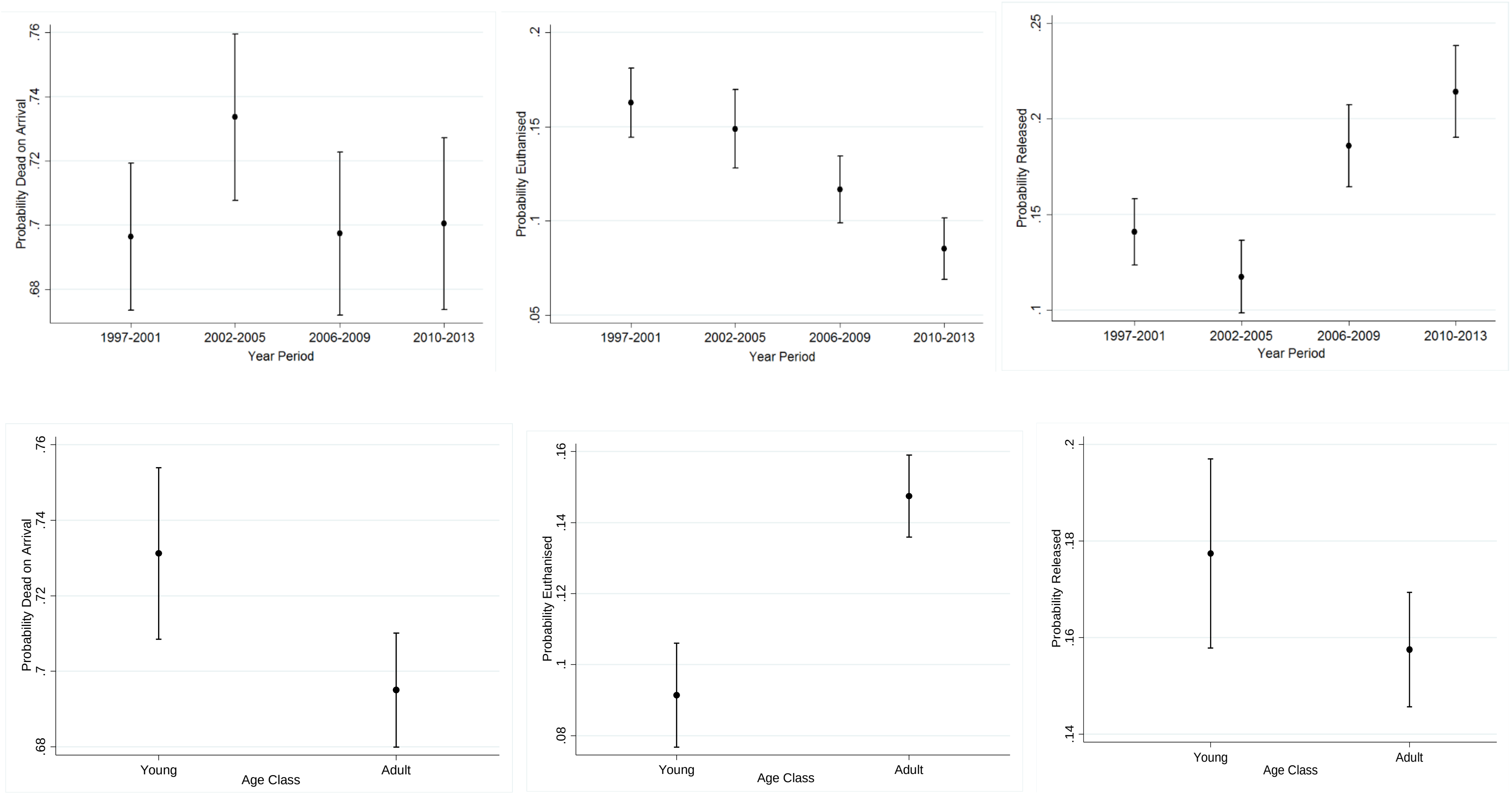

Supplementary Material Figure S2. *Chlamydia*-like signs: Predicted probabilities (with 95% confidence intervals) derived from multinominal regression models of risk factors associated with the diagnostic outcomes (dead on arrival, euthanized, released) for koalas submitted to wildlife hospitals in SEQLD from 1997-2013.

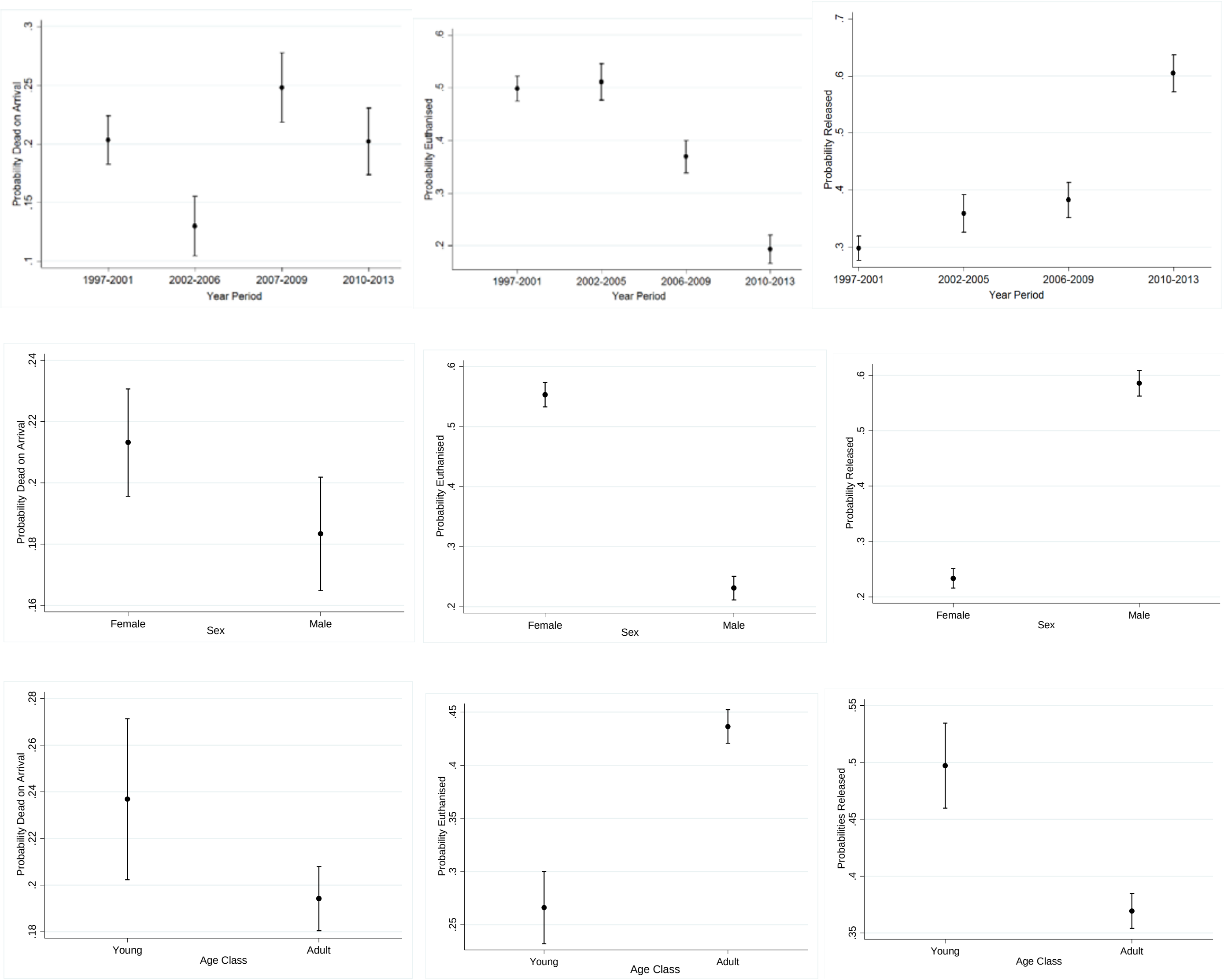

Supplementary Material Figure S3. *Chlamydia*-like signs & wasting: Predicted probabilities (with 95% confidence intervals) derived from multinominal regression models of risk factors associated with the diagnostic outcomes (dead on arrival, euthanized, released) for koalas submitted to wildlife hospitals in SEQLD from 1997-2013.

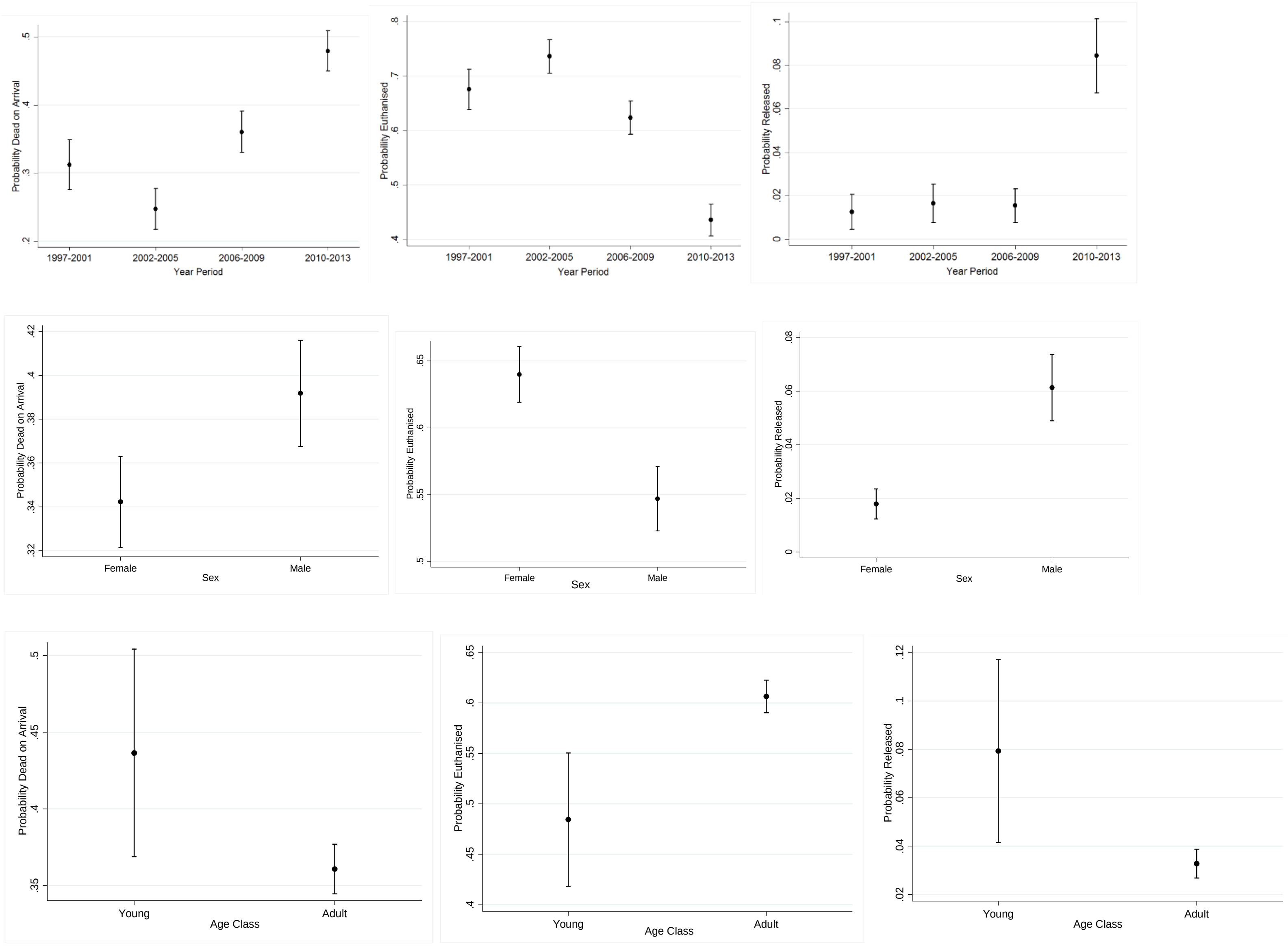

Supplementary Material Figure S4. Trauma by animal attack: Predictive probabilities (with 95% confidence intervals) derived from multinominal regression models of risk factors associated with the diagnostic outcomes (dead on arrival, euthanized, released) for koalas submitted to wildlife hospitals in SEQLD from 1997-2013.

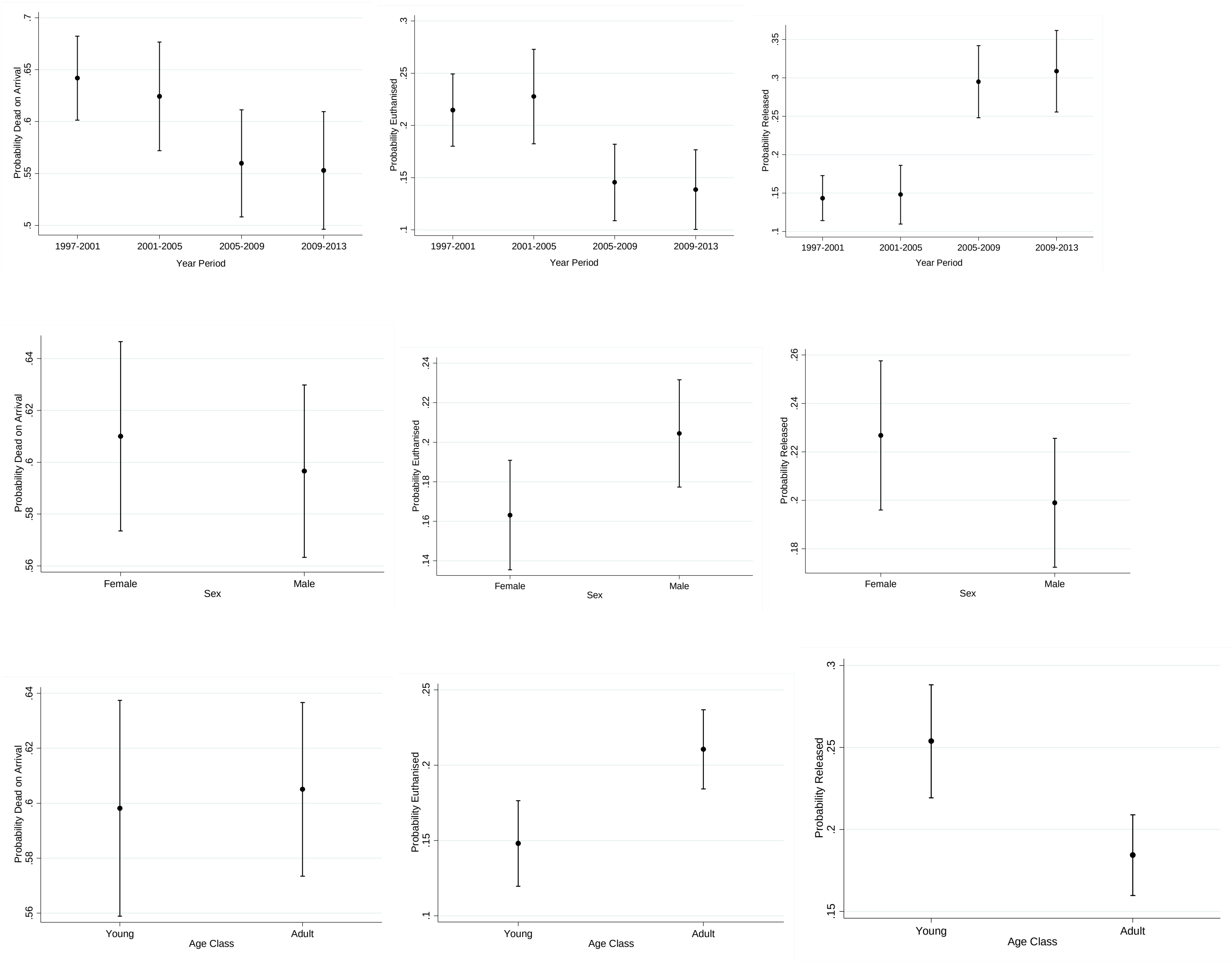

Supplement: Supplementary Material Tables [file srep42587-s1.pdf]
